# 

**Published:** 1857-11

**Authors:** 


VOL. VI. NEW SERIES. No. 11.

### THE NORTH-WESTERN MEDICAL AND SURGICAL JOURNAL.

PUBLISHED MONTHLY.

AT TWO DOLLARS PER ANNUM, IN ADVANCE.

EDITED BY

N. S. DAVIS, M. D.,

AND

W. H. BYFORD, M. D.

NOVEMBER, 1857.

CHICAGO:

"CHRONOTYPE" BOOK AND JOB PRINTING OFFICE.

BARNET & CLARKE, PRINTERS, 189 LAKE ST.

VOL. XIV. WHOLE SERIES. No. 11.

